# Supplementary material for: CHARMM-GUI Covalent Ligand Docker as a Web-based Molecular Docking Platform for Covalent Ligands
Source: bioRxiv. 2026 Jul 16:2026.07.13.738313. Preprint. [Version 1] doi: 10.64898/2026.07.13.738313 (PMC13405148; doi:10.64898/2026.07.13.738313)
Supplement: Supplement 1 [file media-1.pdf]

Supporting Information

CHARMM-GUI *Covalent Ligand Docker* as a Web-based Molecular Docking Platform for Covalent Ligands

Lingyang Kong[†], Donghyuk Suh[†], and Wonpil Im*

Departments of Biological Sciences, Lehigh University, Bethlehem, PA 18015, USA

[†]Both authors equally contributed to this work.

*To whom correspondence should be addressed: wonpil@lehigh.edu.

Table S1. Built-in library of warhead transformations supported by CGUI-CLD.

Warhead Type	Linked Residue	PDB ID	Ligand ID
1-Hydroxy-2,6-Dioxa-3-Oxophosphorinone	SER	1sde	2PB
1,3-Oxazin-6-one	CYS	6mgu	JQS
Acyl Phosphonate	CYS	4xbb	3ZR
Acyloxymethyl Carbonyl	CYS	1nmq	160
Aldehyde	CYS,LYS,SER	6m0k,4pjf,5uzw	FJC,30W,ZPR
Alkyne	CYS	6sgd	LD5
Alpha-Cyanovinyl Carbonyl	CYS	4yhf	4C9
Alpha-Cyclosulfate	ASP	5npb	93Z
Alpha-Hydroxy Sulfonic Acid	CYS	4dcd	K36
Alpha-Pyrone	SER	1k2i	SN1
Amidine	CYS	3i4a	LN5
Aminium Ion	ARG	6b7o	AR6
Aryl Sulfone	CYS,SER	4onm,2pu4	N2F
Aryloxymethyl Carbonyl	CYS	1rwo	KB2
Azide	LYS	2zz6	6AZ
Aziridine	ASP,CYS,GLU	5i23,2gkj,6sxt	66V,ZDR,LXE
Beta-Lactam	CYS,SER	4a52,5kmw	IM2,PNN
Beta-Lactone	GLU,SER	4wv7,4fwg	3UM,SLA
Beta-Sulfanylvinyl Carbonyl	CYS	5w1y	9SV
Beta-Sulfonylvinyl Carbonyl	CYS	6kx3	8ZO
Beta-Sulfonylvinyl Nitrile	CYS	4onn	BY1
Beta-Sultam	SER	5tx9	BSA
Borate	SER	2id8	2DB
Boronic Acid	GLU,HIS,SER,THR	6ert,6vim,2i72,5inh	BVZ,PBC,VA1,6C1
Butadienyl Carbonyl	CYS	3x1i	66B
Carbamate	CYS,SER	5h8i,1uma	N2H,IN2
Carbamide	CYS	6azs	C5S
Carbodiimide	GLU	2db4	DCW
Carbonate	SER	3i2f	DBC
Carboxylic Acid	CYS,LYS,SER	4jap,1htp,1p0i	14U,OSS,BUA
Cyanamide	CYS	6dud,6b88	HB4
Diazo Compound	LYS	3rdh	3RD
Diazomethyl Carbonyl	CYS	2djf	1ZB

Disulfanyl-Ester	CYS	2qnz	DFD
Disulfide	CYS	3orx	1F8
Enamine	CYS	4gpt	51K
Epoxide	ASP,CYS,GLU,HIS	5tng,3ioq,5d6e,5d6f	7XE,E64,57R,94A
Ester	CYS,LYS,SER	4q95,6eyz,1esb	SHV,C5W,BBL
Furan	LYS	1e7u	KWT
Gamma-Lactam	SER	1hv7	616
Gamma-Lactone	CYS,SER	6pzp,6y6u	P7S,ODZ
Haloisocyanide	CYS	6und	QCV
Halomethyl Amidine	CYS	6dge	GBG
Halomethyl Carbonyl	ASP,CYS,GLU	1zrm,5rgm,2vem	BUA,U1D,BBR
Hemiacetal	ASP,CYS,GLU	4ba0,3khu,3rom	5GF,UPG
Hydrazide	CYS,SER	1ayu,5zhr	48Z,KOK
Imidazolidinone	SER	6cl8	MK7
Imidoyl Halide	CYS	6ffm	D8N
Isothiazolinone	CYS	2mlm	2W7
Isothiocyanate	CYS	4ef9	4NF
Ketone	CYS,HIS,LYS,SER	2bdl,1b59,4s2c,1zpz	4PR,2HA,F6R,BUK
Nitrile	CYS,LYS,SER	6rn6,3eww,2i03	K9Q,U1P,AXD
Nitroarene	CYS	6hfv	G1T
O-Acyl Hydroxamic Acid	LYS,SER	6ovz,1scn	N9M,BAA
Phosphate	CYS,HIS,LYS,SER	5wi5,4dwq,2q2t,3txt	0V5,5GP,AMP,DFP
Phosphonohalogenate	SER	4jll	SEF
Phosphorothioate	SER	2xqk	VX
Propargyl Carbonyl	CYS,HIS	6o8i,5zwh	LTJ,9KX
Sulfonyl Halide	LYS,SER	4fi8,3tjm	0UC,PMS
Thiirane	GLU	3i1u	BTW
Thiol	CYS	6r5l	JT2
Thioster	CYS,SER	5gk1,3dpm	2K3,LAS
Thiosulfonate	CYS	1qwz	ETM
Vinyl Carbonyl	CYS,HIS	8pta,5ng1	CIF,ZPN
Vinyl Halide	HIS	9xia	DFR
Vinyl Sulfonyl	CYS,LYS	1m6d,4hjs	MYP,18J

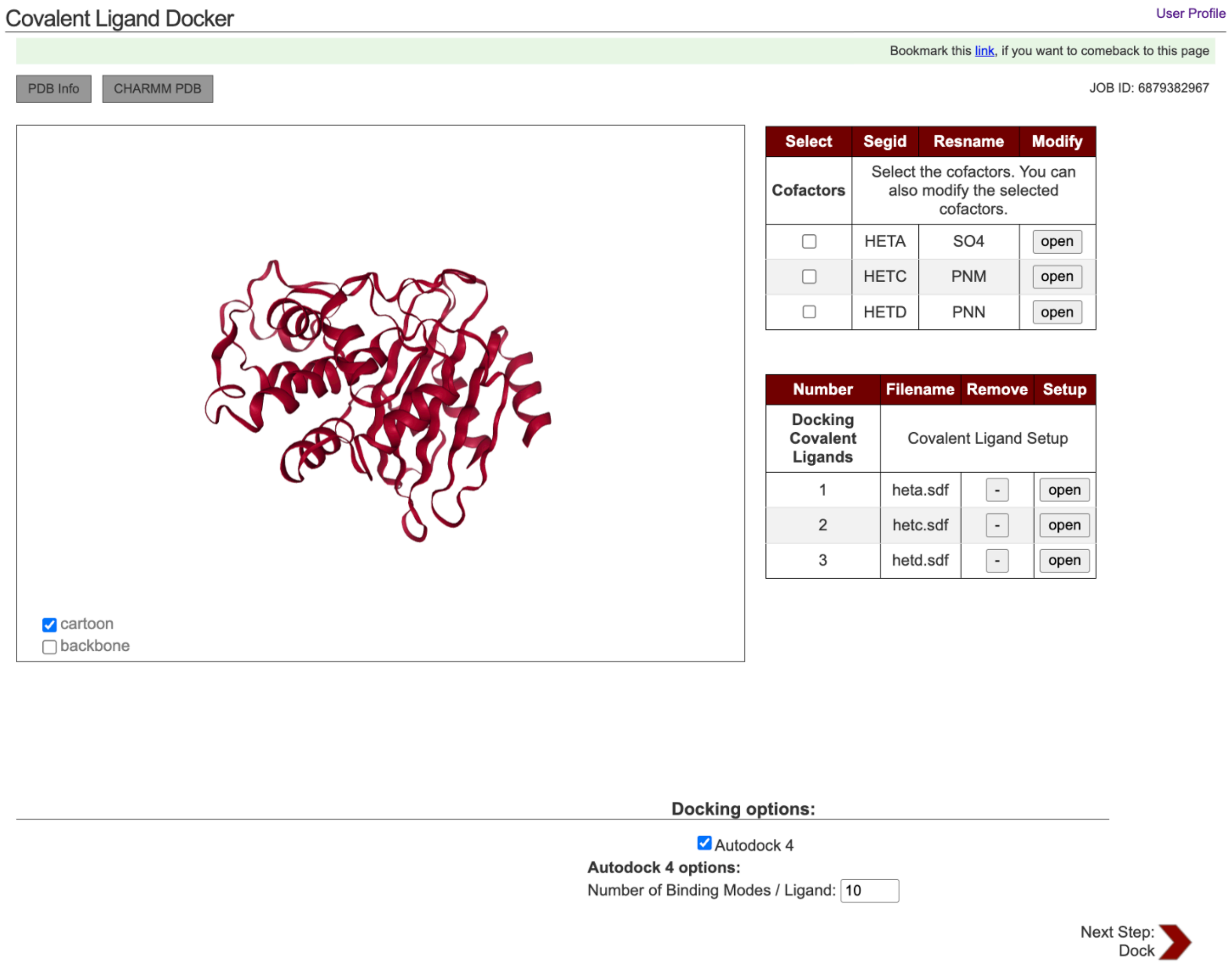

Figure S1. CGUI-CLD setup page for covalent ligands and cofactors.

Covalent Ligand Docker

User Profile

Bookmark this link, if you want to comeback to this page

PDB Info | CHARMM PDB

JOB ID: 6879410103

Docking result:

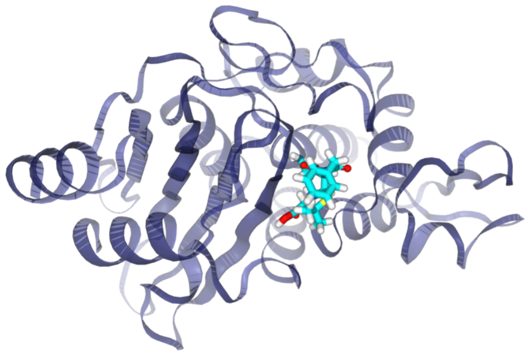

Show All entries

Search:

View	Package	Ligand	Model	Score	Cluster-Rank	RMSD
☑	auto4	hetc.sdf	1	-8.93	Cluster1-Rank02	0.01
☐	auto4	hetc.sdf	2	-8.18	Cluster1-Rank07	0.66
☐	auto4	hetc.sdf	3	-8.93	Cluster1-Rank01	0.00
☐	auto4	hetc.sdf	4	-8.81	Cluster1-Rank05	0.25
☐	auto4	hetc.sdf	5	-8.89	Cluster1-Rank03	0.12
☐	auto4	hetc.sdf	6	-6.68	Cluster1-Rank10	1.97
☐	auto4	hetc.sdf	7	-8.27	Cluster1-Rank06	0.65
☐	auto4	hetc.sdf	8	-8.01	Cluster1-Rank08	0.74
☐	auto4	hetc.sdf	9	-8.86	Cluster1-Rank04	0.14
☐	auto4	hetc.sdf	10	-7.39	Cluster1-Rank09	0.70

Showing 1 to 10 of 10 entries

Transfer Selected Complexes to High-Throughput Simulator

Figure S2. CGUI-CLD result page with 3D visulization, docking scores, and ranking information.

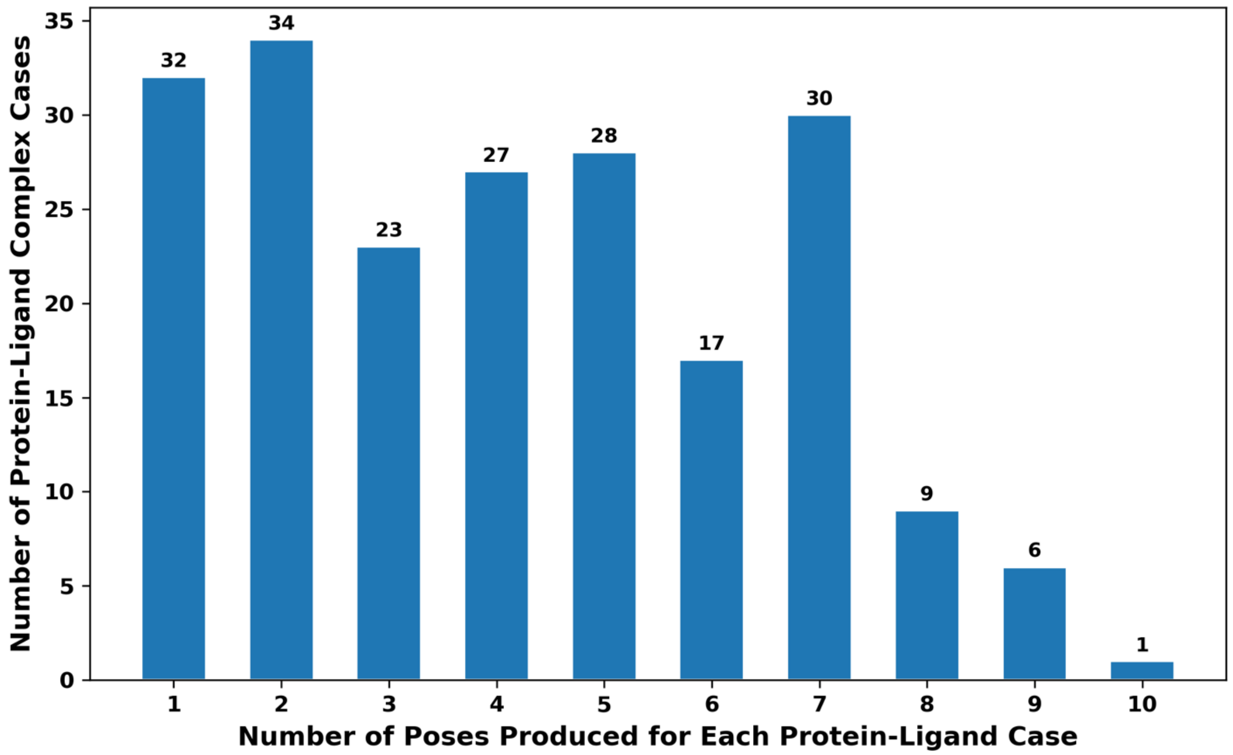

Figure S3. Distribution to show how many protein-ligand complex cases produced how many poses.

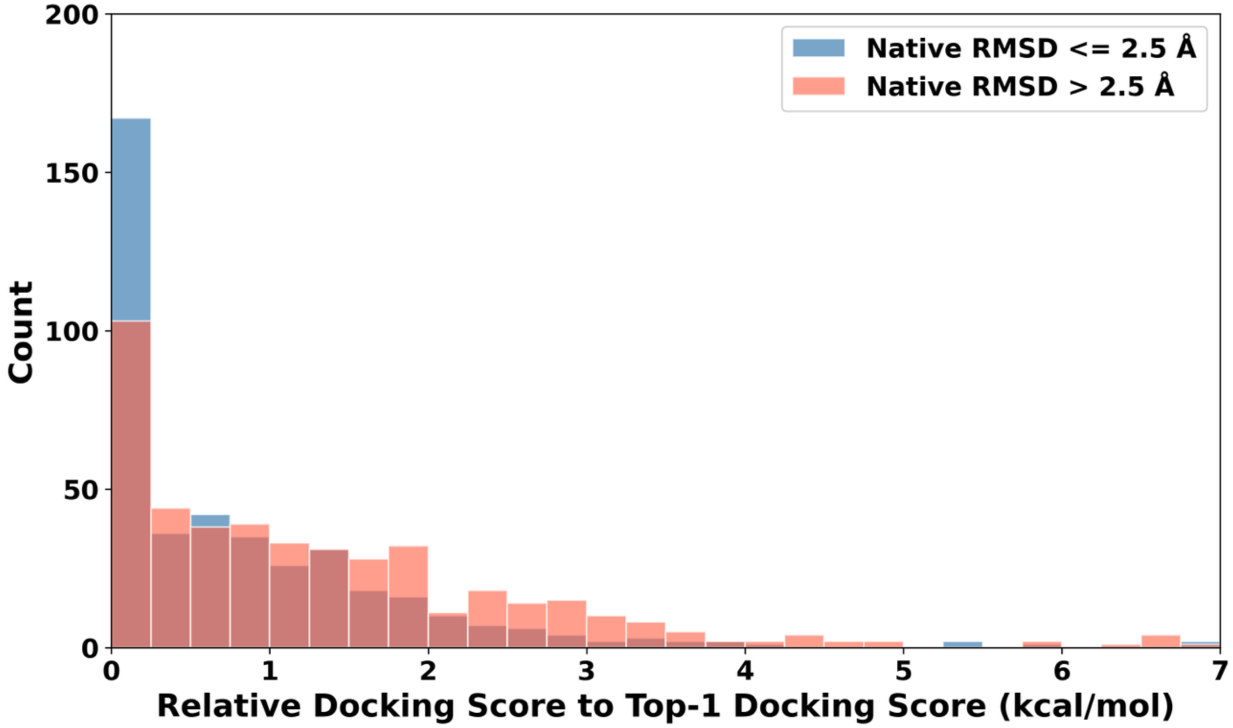

Figure S4. Histogram distributions of relative docking score difference to the Top-1 score in each testcase, covering 865 poses. The ligand poses are categorized based on its RMSDs to their native structures (native RMSD): blue for native RMSD <= 2.5 Å (n=414) and red for native RMSD > 2.5 Å (n=451).
